# Work station learning activities (WSLA) through the ICAP framework: a qualitative study

**DOI:** 10.1186/s12909-022-03794-w

**Published:** 2022-10-31

**Authors:** Judit Sánchez, Marta Lesmes, Clara Azpeleta, Beatriz Gal

**Affiliations:** 1 Facultad de Ciencias Sociales y Educación, Universidad Europea de Madrid, Madrid, Spain; 2 Facultad de Ciencias Biomédicas y de la Salud, Universidad Europea de Madrid, Madrid, Spain; 3 Facultad de Salud, Universidad Camilo Jose Cela, Madrid, Spain

**Keywords:** Active learning, ICAP framework, Medical education, WSLA

## Abstract

**Background:**

Engaging, student-centered active learning activities, such as team-based learning (TBL) and laboratory practices, is beneficial to integrate knowledge, particularly in Medicine degree. Previously, we designed and implemented workstation learning activities (WSLA) inspired by TBL, which proved effective for learning requiring higher-order thinking skills. We now hypothesize that WSLA may also have the potential to be framed into a theoretical model that stratifies learning into interactive, constructive, active and passive modes (ICAP hypothesis).

**Methods:**

An interpretive qualitative research study was conducted to evaluate this idea. Semi-structured interviews were conducted with students enrolled in health science programs after WSLA sessions, consisting of a series of activities accompanying a traditional lecture. Interviews were analyzed according to a deductive approach. Theoretical themes and subthemes driving the analysis were organized around the ICAP modes: passive, active, constructive, and interactive. An inductive approach was applied to provide additional insights.

**Results:**

Students valued preparatory lectures as well as corresponding WSLA activities as highly motivating, especially for the ability to integrate concepts. Although previous research shows that not all activities require high levels of cognitive engagement, students appreciated the opportunity the WSLA provided to discuss and clarify concepts as a group. Furthermore, feedback from professors and peers was highly appreciated, and helped students to construct new knowledge.

**Conclusion:**

In this work, by focusing in understanding the student’s experience, we have evaluated for the first time the WSLA approach in relation to the ICAP model. We found that not only the activity type determines the learning mode, but also the environment accompanying WSLA is a determining factor. Our findings can guide future development of the WSLA approach, which represents an interactive learning methodology with strong potential within the ICAP framework.

**Trial registration:**

Not applicable.

## Background

Active learning is defined by educational researchers as any activity that ‘involves students in doing things and thinking about the things they are doing’, by engaging cognitively and meaningfully with the materials [1], for better understanding of complex ideas and mastering difficult skills [2]. It does not only entail material manipulation but also reflection about the tasks and how they relate to the content [3, 4]. Many studies describe active learning as facilitator of deeper engagement with content [5]. In this sense, many institutions and particularly individual educators do support and implement active learning methodologies, in an attempt to better engage students’ and improve their learning experience [6]. Such approach promotes the development of higher-order thinking skills, such as analysis and synthesis, with major impact on learning [7].

Cognitive engagement is related to motivation and self-regulation [8, 9]. According to Greene [10] motivation is related to students’ engagement with their own learning process and academic performance. She describes deep engagement ‘as involving the active use of prior knowledge and the intentional creation of more complex knowledge structures by integrating the new information with prior knowledge’, while superficial engagement involves rote processing and other intentional cognitive actions that are more mechanical than thoughtful’. Appropriate student-centered active learning leads to greater motivation to engage with learning materials, increased content retention and deeper understanding [11]. Therefore, active learning requires the student to move from passive to more engaged learning states [12]. Given their major impact in Medical Education, devising methodological approaches helping teachers to develop these abilities is essential.

The ICAP framework defines modes of ‘active learning’ according to student behaviors. ICAP is generally considered a theoretical construct of cognitive engagement with a behavioral metric [5]. As outlined by Chi, the ICAP framework establishes four engagement behaviors: interactive, constructive, active and passive. These four engagement styles can be observed by teachers (both teacher-prompted and spontaneous behaviors) while students perform specific activities or tasks [13]. Thus, isolated information storage would be described as passive, while instantiation of previous knowledge would be considered active. Similarly, incorporating new knowledge with previous knowledge is considered to reflect a constructive mode and being able to infer new knowledge, especially in collaboration with peers, is considered interactive [5]. During passive learning, no observable activity can be described; students seem to receive information without any specific action. Some students can, however, actively listening to a lecture by taking notes, summarizing or otherwise showing involvement with materials [14]. In constructive and interactive modes, students generate new knowledge through inferences that go beyond their previous understanding. Interaction amongst students, and its more evolved content-related outcomes, helps to differentiate the interactive from the constructive mode. In the interactive mode, student participation should ideally be as symmetrical as possible to ensure they are actually involved with the material, and thus knowledge is generated collectively. Finally, effective learning is achieved when a constructive and interactive environment promotes quality learning, stimulating cognitive engagement with the subjects taught [15]. Therefore, ICAP is a theory that defines ‘how students can engage with instructional materials cognitively, in an explicit way that is generalizable across learners’ age, content domain and context’ [14].

WSLA has been designed and previously implemented as a new active learning methodology that can be used with large groups of students. It has been implemented within the curricula for the initial years of undergraduate health science courses [16]. WSLA is an adaptation of TBL and works to create integrated learning modules applied to different learning environments, from master classes to gamification and laboratory experiences. WSLA is compatible with other learning approaches, such as case-based learning or inquiry-based learning, so that it is flexible and adaptable to curricular needs. Thus, it may be especially suited to help promote deep and engaging learning by adhering to the ICAP framework.

Very briefly, WSLA is organized around workstations, each one focusing students’ work on concrete outcome(s) within the same clinical case. It follows five different steps which were described in detail by González-Soltero et al. [16]. Over years of experience, we noted that these five steps can be operationally related with the different learning modes mentioned above:

1. Step 1, autonomous work: Students are provided with material for manipulative workshop activities, at least one week before workstation activities, to promote individual readiness and knowledge integration.
2. Step 2, pre-test: Before launching the workstation activity, each participant runs an Individual Readiness Assurance Test (iRAT).
3. Step 3, workstation phase: The professor explains the clinical case and the work to be performed at each station (in teams of four to five students), including details on activity timing and the rotation dynamics between workstations. Here, the teacher acts as a facilitator since the students should be responsible for learning individually and as a team.
4. Step 4, debriefing: This step provides two-way feedback, as the student reports back to the teacher about what they learned and, in turn, the teacher provides the student with comments favouring active and constructive learning.
5. Step 5, final evaluation: Set by the teacher, this evaluation can be done individually and in groups, but it always scores knowledge of the subject. The final evaluation includes a 50% pre-test score, 25% the WSLA group score and 25% post-test score.

WSLA has been shown to improve student academic performance specifically for those learning outcomes related to higher order thinking skills [17]. Here, we hypothesized that the WSLA may have the potential to be framed into ICAP model. Thus the ICAP hypothesis is used to provide a theoretical foundation which guides our study, similarly to the use of Bloom’s taxonomy by other authors [18]. To further investigate this possibility, we planned our qualitative research study with an interpretive methodology [18] and conducted semi-structured interviews with students enrolled in Health Science undergraduate WSLA programs. We used their reported experiences in lieu of the overtly observed behaviours, as described by Chi and collaborators. Our qualitative study aims to discuss and interpret the educational implications of the WSLA approach within the ICAP hypothesis. By framing the WSLA steps within ICAP, we intend to enhance our understanding of student learning processes.

## Methods

The present qualitative study has been planned and conducted following pragmatic approach principles [19]. While this positioning is common in mixed methods studies, our study was aligned with the pragmatic principles that focus on the needs of the research question. Deeper theoretical positioning will not be discussed here as it would exceed the objectives of our study, as is usually the case in applied disciplines, particularly in Health Science education [20]. Thus, our study is interpretive in nature [18], as we seek to understand student´s experience (via semi-structured interviews) and to relate it to a learning framework, such as the ICAP. This process requires the researcher’s interpretation of the data, as Thorne suggests, supporting our interpretative approach.

To run this study at Universidad Europea de Madrid, students were purposely selected from a variety of Health Science degree programs [21]. The reasons for adopting a purposive strategy were based on the assumption that, given the aims and objectives of the study, specific kinds of people may hold different and important views about the ideas and issues at question and therefore need to be included in the sample. Semi-structured interviews (academic year 2018–2019) were planned and conducted by two of the authors. Professors external to this study were responsible for selecting students meeting a combined profile (low achievers with high and low motivation and high archivers with high and low motivation) using a checklist (motivation/achiever). Motivation was defined by rate of course attendance and class participation, while course work completion and midterm and final exam show up rates (course attrition) defined achievement. Work consists of a portfolio of different activities, which were evaluated with different rubrics. Learning results associated to acquisition of skills were evaluated through a practical laboratory work. Multiple-choice was the main evaluation used in the midterm and final exam. Work completion weighs 40% and midterm and final exam weighs 60% of total subject mark achieved.

Students meeting the checklist criteria, who also experienced WSLA, were invited to participate and those who gave their consent were included in the study. This resulted in a group of 12 students: 5 students from the Medicine program (identified with M code), 3 students from Physiotherapy (P code) and 4 students from Dentistry (D code) (Table 1). The aim was not to let the analysis be driven by students’ profiles but to collect diverse views and to infer the type of ICAP engagement the WSLA provided to them. International students were included but neither nationality nor gender were considered for the analysis (see details in Table 1).

**Table 1:** Students profile

	Interview criteria				
**Student**	**Code**	**Motivation**	**Achiever**	**Gender**	**Nationality**
**Medicine 1**	M1	High	High	Male	Latinamerican
**Medicine 2**	M2	High	Low	Female	European
**Medicine 3**	M3	High	High	Male	Latinamerican
**Medicine 4**	M4	Low	Low	Female	European
**Medicine 5**	M5	High	Medium	Female	European
**Physiotherapy 1**	P1	Low	Low	Male	European
**Physiotherapy 2**	P2	High	High	Male	European
**Physiotherapy 3**	P3	High	Medium	Male	European
**Dentistry 1**	D1	High	High	Female	European
**Dentistry 2**	D2	High	High	Female	European
**Dentistry 3**	D3	High	Medium	Female	European

Two of the authors, who had no other form of relationship with students, conducted semi-structured interviews (40 min each), which were recorded and transcribed. A mainly theoretical deductive approach was used as a key analytical strategy. Deductive themes/subthemes were defined by relevant literature predominantly described in the ICAP framework: the passive, active, constructive and interactive modes (Table 2). Examples supporting these modes were identified in the transcription of the interviews and most relevant verbatim were selected. Additionally, our strategy to start from a theoretical theme (i.e. ICAP) while being open to inductive themes is considered as a form of thematic analysis [22]. Inductive themes are those not defined prior to the study, but recognized as relevant during the analysis [22]. Deductive and inductive subthemes and, most importantly, the relevant verbatim were all included in the results and discussion of this paper. Subthemes were defined from the literature within each theme, and codes and verbatim were selected for reporting (see theme, subtheme, and codes in the Table 2).

**Table 2:** Deductive themes and subthemes

Deductive themes				
**Theme**	**Student commitment/participation**			
**Subthemes**	**Passive**	**Active**	**Constructive**	**Interactive**
**Codes**	Traditional lectures	Student -centered	Self-directed learning	Able to understand an original idea based on an interaction with their peers
	Teacher centered	Manipulation	Self-explanation	Interacts with the teacher
	Surface learning	Integration	Reflects on lessons learned	
		Retention	Deep learning	

Ethics approval and consent to participate was obtained (see Declarations). No statistical method was applied since this is a qualitative study. No power calculation was performed to predefine the group size, which follows the typical design of interpretative qualitative research.

## Results and discussion

Given the interpretative approach of this qualitative study, we herein report and discuss results from semi-structure interviews to provide a thorough assessment of the students’ perspective regarding WSLA and ICAP. We separate reporting of deductive and inductive themes to ease interpretation and analysis.

### Deductive themes

Four deductive subthemes were analyzed from semi-structured interviews: passive, active, constructive and interactive.

#### Passive

We found that students rated positively traditional lectures prior to WSLA. They believed they were necessary for providing knowledge of the content prior to the WSLA activities. In fact, in courses that combined traditional classes with PBL methodologies, students tend to rely on lectures [23]. In this sense, interviewed students stated:

**M3:** ‘What I really liked was that in class they usually presented the content they were going to ask about later in the activity (…)’.

Usually, traditional classes are structured in the passive mode. In spite of this, as Chi and Wylie [5, 24] already noted, listening to the professor during the lecture previous to WSLA can actually entail an active process:

**D1:** ‘I think the theory, the classroom part of it is important as well. I think it gives you a base knowledge to then build upon in the labs. I think without that base knowledge you would not be able to squeeze as much out of the lab as you can with the classroom’.

It is also interesting to observe that traditional class ratings were intimately linked to their perception of the teacher (teacher-centered). This is logical in the passive mode, as professor structures the classes based on their own perspective [24]. In this sense, students claimed that a good professor is one who directs their learning, and offers their own perception and experience:

**M4:** ‘You learn so much more when the professor guides you and tells you (…) Then, everything turns out much better in the end (…) based on repetition and seeing the material, but I think that it doesn’t make sense for them to give you a list of instructions and say: ‘Figure this out… With the stations you were assigned.’ If no one tells me whether or not I got it right, there’s no point’.

**M3** ‘I’m pro lecture. What I like about lectures (…) is that you not only get the professor’s perspective – which is always great – they also always go on slightly off-topic tangents. They tell you about their own experience and such, and if they don’t, they help structure the material. You might have all the information in the book, but the professor puts it in order (…) So, I would certainly recommend having a lecture before one of these activities. I mean, I don’t think they should be a replacement, but rather an additional activity (…)’.

Students also felt lectures are not enough for effective learning, suggesting they understand that traditional classes need to be complemented. In fact, they stated that, in order to acquire long-lasting learning and deeper knowledge, they need teaching that supplements lectures. Importantly, as can be noted from our interviews and other published studies, students do not share negative opinions held by innovative teachers on lecture-based classes [24].

Defining the learning process is a common educational goal. In 2003, Entwistle [25] published a paper researching professors’ and students’ conceptions of teaching-learning. He described a model in which the different learning concepts correlated with different approaches toward studying and the level of understanding they achieved. Learning can be understood as a reproduction of the content being taught, a view that is associated with content-guided and teacher-centered learning. If teaching is student-centered and geared toward learning, then it is understood as a process of transforming information based on students’ own understanding and prior knowledge. How can we cognitively involve students in their own learning so that it is indeed transformative and meaningful? We address this question in the next section.

#### Active

Students’ behavior and their degree of commitment to certain activities while they are learning can be considered as active learning. Based on this model, we next analyzed whether student perception about WSLA was helpful for generating meaningful knowledge. We first researched whether the active mode was present during the WSLA activities, by shifting away from a traditional content- and teacher-centered surface learning. Thus, we focused analysis on five dimensions: balance of power in the classroom, the function of the course content, the role of the professor and students, responsibility over learning, and the purpose and process of assessment. Two of these dimensions were vitally important to our study: learning accountability and the role of professors and students [26].

With regard to students’ responsibility for their own learning, they believed that:

**M5:** ‘(…) the student has to put in the majority. I don’t know, maybe it’s about 80%-20%.’

**D1:** ‘I think you learn better, even if you get it wrong, you learn. You learn better from your mistakes or from where they could have come. By the end of the lab you never leave confused, you know, by the end of the lab the teacher took you through what the answer was, what you were supposed to do.’

Student-centered learning is put into practice in the various active methodologies [26], as in WSLA. Some students found the independence associated with this accountability to be rewarding, and they valued this positively during WSLA activities:

**D1:** ‘The way that they do it never leaves you confused, just gives you the independence to figure things out on your own initially, and then at the end you’ve learned what you are looking for. I find this independence is nice.’

While literature supports the importance of students’ accountability, the revision done by Weimer [27], indicates that students may be not mature enough to be given full responsibility, and they let teachers take the lead. This is consistent with our students’ belief that traditional classes are necessary before WSLA activities. Importantly, Chi and Wylie [5], deemed physical manipulation of educational material, including ‘taking notes’, as hallmarks of active learning mode. When we asked students about manipulating and interacting with materials during WSLA activities, they confirmed that they have a positive perception, as it helps them better understand the content:

**M2:** ‘I love class, because I get a lot out of it. I have all the things discussed in my notes, because if you open the presentation and didn’t go to class, at least I’m like, ‘Oh my god! Why is that? Check the book, but it doesn’t say the same thing. What are you supposed to do?’ Having notes, having the explanation, is like going. ‘Obviously, it’s so obvious (…)’.

**F1:** ‘Theory is just theory, but when you put it into practice, you see it and it becomes so much clearer. (…) everything you do practically on your own sticks with you so much more.’

**F3:** ‘While if you do it and see it, I think the concepts stick with you so much better.’

**O2:** ‘Lab is better because at classroom we don’t have the material for doing the activities.’

**M5:** ‘I like the practicums. We do things so you can actually see them. They come up to you and explain, ‘This is how pulsis works; take a look at the blood, which type is it?’ These are things that are really memorable.’

However, while students may show these active behaviors, it does not necessarily mean that significant changes are occurring at the cognitive level, as we also confirmed in the verbatim:

**F1:** ‘I’m talking about something like my last degree program, where you would go to the lab and they’d say, ‘make aspirin.’ Something really traditional. You follow the instructions and afterwards, what have you learned? Nothing, because it’s like making cookies. You’re following a recipe: ‘I did this, this, and this. Hey what did you do? I don’t know, let me check the recipe’.But if the interaction is: ‘What would you do or how would you do it’ your brain has to work and if you do it, you understand so much more.’

In fact, these situations are commonly found in most science practical activities. Students are given to follow a specific laboratory protocol, but that doesn’t mean that they are integrating or applying the relevant concepts [28]. In this sense, methodologies such as WSLA may provide students with the opportunity to design activities in collaboration with others and with feedback from the professor.

Finally, we found that the active mode stands out by helping students to retain information better and to integrate new knowledge, thus achieving meaningful learning. In supporting this, students stated that WLSA activities helped them significantly:

**M1:** ‘In the best way, I mean, the idea is to really understand it because knowing something for a test is one thing and actually learning it is something else entirely. I really think that – in my particular case, of course – integrated activity did help me. I remember a lot.’

**M5:** ‘I really like it because it helps me lock down the information and not just memorize something, regurgitate it on the exam, and forget it (…)’.

**D1:** ‘I think a 100% it’s far more superior than a classroom experience. I think you need the classroom experience to gather the information beforehand and they also make sure that you get the most out of your labs, but the lab I think is very, very, important in your learning and I haven’t forgot anything that I’ve learned in the labs so far, because you are physically doing and you learn as you are going along and I personally hadn’t had a lab experience in my previous studies, it has always been theory-based and the lab is something that I enjoy the most; it backs my understanding more than anything else, and yes, I remember it more than anything else.’

**M3:** ‘Since I really like to analyze what I’m doing, I felt like I internalized everything so much better’.

In summary, by permitting physical manipulation of material within the learning environment, we conclude that WSLA provides better understanding of the content and more effective retention and integration of new information in knowledge from lectures. These general student´s perceptions are supported by previous work published by our research group, as we described that students understand better those learning objectives associated with higher levels of cognitive learning in Bloom’s taxonomy in the context of WSLA methodology [16].

#### Constructive

Constructive learning implies that learners generate new ideas beyond what is provided in materials. Self-explaining, for instance, is an ability of the constructive mode. To promote this mode, learners need articulating what a text, a sentence or a physiological process should mean to students by promoting interpretations that are not explicitly stated, or by providing them an argumentative explanation. Both the inferences and the interpretation the students make from this process should go beyond the information provided. If the students’ self-explanation is just repeating what they read, then they are not self-explaining constructively, because no new information is provided. Thus, we included in the analysis both independent study and self-explanation codes to deepen into the constructive mode.

We found that students believed that prior independent work was vital for WSLA activities:

**O1:** ‘(…) I always prepare quite a bit for labs, because I like to feel prepared and be able to participate and understand even more. I never go having done zero prep work, and when you’ve already taken anatomy, histology, physiology, and the biology you need to know to be able to internalize everything, when you’ve already studied the theory, I think it’s great to internalize it in this way (….).’

Jones and Edwards [29] evaluated the efficiency of biology students getting ready before a laboratory session. They demonstrated that better preparation led students to better performance, as well as better compliance with professor-designed guidelines, including time management. Thus, not preparing for WSLA activities requiring prior knowledge may result from poor self-directed learning abilities, as shown before [30], which relates to Weimer’s claim on some students being unable to direct their own learning [27]. In 2011, Abraham [31] argued that self-directed learning requires a preparedness dependent upon students’ personal attributes. They concluded that, although first-year medical students taking physiology seem to be prepared for self-directed learning, specific strategies to improve time management skills should be implemented [32]. In this sense, the self-directed learning-based design of WSLA, and assessment using a pretest, require preparedness for the activity.

In an ICAP framework, self-explanation is part of constructive learning that leads to new understanding. Another code of interest found in the verbatim within the constructive subtheme was self-explanation:

**M2:** ‘(…) practicums force me to explain it; I’m one of those people who when they explain it, they know it. Even if I wrote it down and understood it, if I don’t explain it and really see that I understand it, I don’t. Practicums force me do that.’

The constructive learning mode also requires students to synthesize their own ideas and create something new, beyond what was provided in the material [5, 33]. Weimer [27] claimed that in order to promote constructivism, the subject content itself should entail students with learning to learn competences. We found this is something achieved by WSLA methodology:

**D1:** ‘I’m learning again how to learn’.

**M5:** ‘because you might not know it very well at the time, but then you check your notes and say: ‘Oh! I did this in the lab, obviously, and this was the part that stayed the clearest, and this one did that,’ or: ‘This is the thing that didn’t turn out right’.

The process of reflection involves students’ assessment of what they do and do not understand over the learning process. That reflection means this knowledge can be applied in different contexts, entailing interpretation and generation of new understanding. Thus, according to the verbatim, we may conclude that students noticed that reflection process after WSLA activities. Interestingly, the verbatim also suggested students felt they learn best when they use the WLSA methodology as it may generate deep learning:

**M1:** ‘In the best way, I mean, the idea is to really understand it because knowing something for a test is one thing and actually learning it is something else entirely. I really think that – in my particular case, of course – integrated activity did help me. I remember a lot.’

**M2:** ‘I really learned a lot with the integrated activities, and you’re not all that aware of it until you take the exam.’

**M5:** ‘And I think that’s the problem: they don’t understand things, they’re stuck with the traditional method, which is understandable, it’s been done for so long, but I really think that the workstations helped me to say: ‘Okay, M5, time to shift the mindset. You can’t just memorize it and regurgitate it because you just can’t. That time has passed’.

Although the concept of deep learning in higher education is not new, in order to better interpret these statements, it may be important to clarify what surface and deep learning means from an historical perspective. When Marton introduced the concept, he proposed learning has to be described in terms of the content that is learned and so described two levels of learning processing: the superficial and the deep [34]. For example, in Physiology teaching, the superficial approach would describe the facts of a physiological process. Instead, a deep approach would focus on the mechanisms. Biggs presented a theory of learning based on the importance of students becoming aware of their own learning processes [35]. This yielded to the concept of meta-learning as a model in which the relationships between personal factors, the situational context and the quality of the results are all mediating the learning capacity of the students. Thus, learner-centered activities more likely take students to deeper levels of understanding [36, 37], as it occurs in WSLA.

As shown above, we found all these reflections in the verbatim. As already noted Biggs, Entwistle. Ramsden and Tagg [25, 35, 38, 39], students generally agreed that deep learning is represented by a personal commitment to understand the material. Students using ‘surface-level processing’focus on the substance of information and emphasize memorization techniques [35, 38]. In contrast, students using ‘deep-level processing’ look for the underlying meaning of the information. We found evidence of this in the students’ statements regarding the use of various strategies such as reading widely, combining a variety of resources, discussing ideas with others, reflecting on how individual pieces of information relate to larger constructs, and applying knowledge in the WSLA situation. Another characteristic of deep learning is integrating and synthesizing information with prior learning in ways that become part of one’s thinking from different perspectives [10]. In fact, this approach is quite well embedded with the ICAP which has been used as a framework in our research. Thus, when students spend a prolonged amount of time on an activity that may seem challenging, they are required to work independently beforehand to then integrate knowledge with that emerging from the workstations, leading to deep learning and higher-order thinking.

#### Interactive

Finally, reflections about the interactive learning mode were also extracted from our analysis. WSLA workstation activities are designed for students to discuss the learning outcomes worked on in each session. In order for the discussion to be effective, these conversations must spark an interactive debate. The quality of student conversations increases in interactive learning when they have done self-directed tasks prior to the activity [39]. In most cases, the substantiality of the conversations will therefore be directly proportional to self-directed learning. We found evidence of the constructive mode of learning in the students’ verbatim:

**M2:** ‘(…) For example, one person understands the theory perfectly, sees the question and says, ‘It’s this, and another person disagrees saying: ‘No, because it might actually be that’ and it gets really convoluted. Another person says, ‘Well, we should do it like this, and another one goes, ‘Explain it to me’. So, even though it may not seem like it, in the end we all have such a better understanding. We see all the possibilities of how it could have gone and why x wasn’t the answer, it was y. You really understand it so well’.

As defined by Chi and Wylie [5], we considered an interactive learning mode when the following two criteria were met: (a) dialogs between students must be constructive, so that new ideas should stem from these dialogs, and (b) all students should contribute to discussion so that ideas emerge at the group level. According to the verbatim, students felt that this form of interaction improved their understanding, so that learning is transformative and meaningful. Along this line, a number of authors including Wells [40], have focused on the language of these interactions as a central part of the teaching-learning process. Some of these principles of interactive mode can be extracted from our students’ comments:

**M4:** ‘I think that it’s really important to work with a group like you: that wants to learn; I really felt that when I was able to choose my group, I mean, I pick the people I want to work with (…) generally they’re people who are there to learn and get something out of the practicum (…) when you work in a group, everyone explains their ideas. You might feel very strongly about your viewpoint, but when you see it from someone else’s you go, ‘Wow! I hadn’t noticed that, You know? It’s not just the work at the workstations and that they make you create a thought process, but also that you see your classmates’ ideas and say, ‘I never would have thought of it that way. That never would have occurred to me’.

**M3:** ‘Something that I really liked is when we were separated into groups. That encourages teamwork, because you’re not only doing the activity, but also exchanging information with others and seeing what they think. I think that’s really valuable.’

**D1:** ‘When it comes to learning and retaining information I find that individually I would second guess myself more and I would not be 100% sure of what I was getting right whereas if I was in a group, people look at the same information from different angles and I think that’s important in retaining your information as well, when it comes to retaining information group work is maybe a little better.’

In agreement with ICAP, we found that students consider that learning slows when these dialogs are not equitable or constructive at the group level:

**F1:** ‘Your classmates are more important than the professor, because it’s not the same to have a classmate who doesn’t care, who’s just going through the motions, and one who really goes, ‘Hey! Check this out! .

**M3:** ‘There are people who don’t take the activity as seriously and, since we’re in groups, that creates space for dispersion among groups. Then people are like, ‘Um, I don’t know what to do,and they waste time (…)’.

**M4:** ‘(…) I think it’s really interesting, but only when there are people who want to participate, because you can have people who say, ‘Okay. Let’s just fill in the paper and go home’.So, it’s really important in my opinion.’

Another important aspect of the interactive mode is that students appreciate that conversations with their professors are enriching [33]. In fact, the final phase of the WSLA methodology, the debriefing, targets this aspect specifically:

**M2:** ‘Yes, the way I remember it, the entire class was led by the professor. And that’s also great, because when you’ve made an effort to learn something and suddenly understand it and you share it with the whole class it’s like, ‘Wow! I can do this’.

**M3:** ‘Then each student has the opportunity to share and say, ‘Look, I think it’s this,’ and then you debate the differences of opinion and stuff, and it’s really nice, and the professors answer questions (…)’.

**F2:** ‘I love the discussion. I really like the conversation.’

Here, feedback can be understood as a corrective tool, in which the teacher holds the position of expert by providing information to a passive recipient (the student), or it can be considered a tool for guidance wherein the professor also learns from the student through dialog and shared experiences guiding them towards meaningful knowledge [41]. This approach is in line with the interactive framework, as feedback implies comments and suggestions to allow students to make their own revisions and the dialog helps them generate new knowledge [42].

Taken together, our assessment of students’ perceptions confirms that WSLA is positive for their learning when dialog is interactive, constructive, and equal for all group members. Thus, the WSLA methodology provides the necessary framework to promote the student’s engagement with the learning material, through equitable dialog established student-to-student and student-to-professor, based on which more meaningful learning flourishes.

### Inductive themes

Having reported results obtained from the deductive themes, we now describe data extracted inductively. Three inductive themes spontaneously emerged from the interview analysis: environment, time and applying the knowledge. Since these themes were not initially integrated in the design, we consider useful reporting how many students supported each of them to provide a better assessment of their relative importance.

### Environment

With regard to the learning environment, 5 out of 12 students perceived different learning environments based on the WSLA they participated. Students associated traditional classes with a learning environment, and WSLA with another context. Everything entailing a physical interaction with the WSLA material was associated with laboratory work, as these students stated:

**M2:** ‘I really like my classes and I almost prefer that the integrated activities are integrated laboratory activities, because we have a mental separation of spaces: class is class, and we need to be quiet there, and it goes from this time to that time, and if we have a practicum, it’s a practicum, so we have that dynamism, our routine, our roadmap, etc.’

**D1:** ‘Somehow it does make a difference being in the lab.’

The learning environment can be defined as the workload, the methodology, and the structure of the course [6]. It is correlated with academic performance and learning effectiveness. Various studies have confirmed that the student learning results and their own learning strategies are correlated with the student perceptions [43, 44]. Aghamolaei et al. [45], claim that ‘the educational environment influences how, why, and what students learn’ and they argue that the physical environment is one of the main determinants of the learning process. Critically, the learning environment influences the student commitment to the material, their motivation, and outcomes [7, 46–48]. Students perceive the environment as a determining factor for their own behavior [49], while Modell [50] posits that active learning environments need to be simulated so that students get involved in their own learning. This is exactly where WSLA seems to act to provide with environmental frameworks for the students to learn actively. The analysis of the verbatim effectively confirms that the WSLA environment was decisive for their achievements.

### Time

Time was another theme emerging spontaneously in the verbatim in 2 out of 12 students:

**M2:** ‘(…) I think it is important to prepare beforehand, because we don’t have all the time in the world and we shouldn’t, to go back to the drawing board and try to understand it from the beginning. I think the work done before the practicum is also very important to ensure we really make the most of it.’

**M1:** ‘Integrated activities don’t have much time for students to really figure out each and every one of the workstations (…)’.

From these statements, it can be surmised that the students who failed to properly prepare themselves before WSLA might waste a lot of time trying to connect the new information with prior knowledge. That would result in deviating their attention from what the WSLA is actually offering [29]. Weimer [27] suggests that course content should not be treated as a goal, but rather as the vehicle for students to learn how-to-learn and to develop time management and study skills, among other things. Thus, this suggests that the professors running WSLA activities should be very explicit for the need of preparation. Remarkably, this theme is associated with the constructive commitment mode, where self-directed learning done before sessions is crucial.

### Applying knowledge

The third inductive theme that arose from the interviews is applying knowledge. Six out of 12 students discussed the importance of both receiving theoretical content and applying it subsequently in a laboratory practicum for more effective learning:

**F2:** ‘I remember the theory because I studied it, but as time goes by I have to think about it to remember, while the practicum material is more ingrained.’

**F3:** ‘I understood the theory we looked at because I was able to apply it.’

Anwar [46] recognizes the effectiveness of including a variety of sessions and teaching techniques to capture attention. In fact, the different learning environments (classroom, laboratory) should be designed based on learning outcomes. For their part, students need to build higher-order thinking models to gain a deeper understanding of the material. A practicum is more appropriate for higher-order thinking outcomes requiring the application of knowledge to a real-life simulated situation as it is the case for the WSLA session. That is why one effective alternative is to use multiple teaching and learning approaches to ensure they keep students motivated and interested [51].

Importantly, we also found students valued background knowledge:

**O1:** ‘I think I prefer having at least some background knowledge so I can formulate ideas and have a better understanding of how it works. That’s my preference, but I don’t think it’s bad for you to learn those things straight from the lab.’

**M2:** ‘I think there needs to be a time when I’m taking notes listening to the professor without my computer, and then the practicum. I think we need both, because leaving us all on our own is very, very risky.’

We found this inductive theme to be strongly associated with the constructive and the integrative mode of learning discussed as part of the inductive approach.

## Conclusion

Using a qualitative approach, we confirmed the hypothesis that WSLA activities may be framed within the active, constructive, and interactive modes of the ICAP model. Remarkably, an inductive analysis allowed us to conclude that not only the activity type determines the learning mode, but that the environment accompanying WSLA is a determining factor. Thus, by controlling the learning environment, student characteristics and the specific and general organizational aspects of WSLA sessions all may lead to deep, transformative, meaningful basic science learning for Health Sciences students. Our work thus highlights the educational strengths of the WSLA approach framed within ICAP, which can be exploited to promote active, constructive and interactive engagement of students with their own learning process.

Finally, it is important to note that one limitation of our study is that it relies mostly on students’ testimonials after WSLA was practiced. Future research may thus include structured classroom observations during activities designed with the WSLA methodology, to provide additional evidence of the educational value of framing WSLA in the ICAP framework. In fact, we have already obtained some preliminary data that point out those students who interact more and in a symmetric way, are those who perceive they learn in a deeper and meaningful way.

## List of abbreviations

ICAP: Interactive, constructive, active and passive
WSLA: Workstation learning activities
TBL: Team Based Learning
